# Increased Recruitment but Impaired Function of Leukocytes during Inflammation in Mouse Models of Type 1 and Type 2 Diabetes

**DOI:** 10.1371/journal.pone.0022480

**Published:** 2011-07-25

**Authors:** Ulrika Sofia Pettersson, Gustaf Christoffersson, Sara Massena, David Ahl, Leif Jansson, Johanna Henriksnäs, Mia Phillipson

**Affiliations:** Department of Medical Cell Biology, Uppsala University, Uppsala, Sweden; Los Angeles Biomedical Research Institute, United States of America

## Abstract

**Background:**

Patients suffering from diabetes show defective bacterial clearance. This study investigates the effects of elevated plasma glucose levels during diabetes on leukocyte recruitment and function in established models of inflammation.

**Methodology/Principal Findings:**

Diabetes was induced in C57Bl/6 mice by intravenous alloxan (causing severe hyperglycemia), or by high fat diet (moderate hyperglycemia). Leukocyte recruitment was studied in anaesthetized mice using intravital microscopy of exposed cremaster muscles, where numbers of rolling, adherent and emigrated leukocytes were quantified before and during exposure to the inflammatory chemokine MIP-2 (0.5 nM). During basal conditions, prior to addition of chemokine, the adherent and emigrated leukocytes were increased in both alloxan- (62±18% and 85±21%, respectively) and high fat diet-induced (77±25% and 86±17%, respectively) diabetes compared to control mice. MIP-2 induced leukocyte emigration in all groups, albeit significantly more cells emigrated in alloxan-treated mice (15.3±1.0) compared to control (8.0±1.1) mice. Bacterial clearance was followed for 10 days after subcutaneous injection of bioluminescent *S. aureus* using non-invasive IVIS imaging, and the inflammatory response was assessed by Myeloperoxidase-ELISA and confocal imaging. The phagocytic ability of leukocytes was assessed using LPS-coated fluorescent beads and flow cytometry. Despite efficient leukocyte recruitment, alloxan-treated mice demonstrated an impaired ability to clear bacterial infection, which we found correlated to a 50% decreased phagocytic ability of leukocytes in diabetic mice.

**Conclusions/Significance:**

These results indicate that reduced ability to clear bacterial infections observed during experimentally induced diabetes is not due to reduced leukocyte recruitment since sustained hyperglycemia results in increased levels of adherent and emigrated leukocytes in mouse models of type 1 and type 2 diabetes. Instead, decreased phagocytic ability observed for leukocytes isolated from diabetic mice might account for the impaired bacterial clearance.

## Introduction

Type 1 and type 2 diabetes (T1D and T2D), characterized by long-term hyperglycemia when untreated, are both inflammatory conditions. Islet inflammation promotes β-cell destruction in T1D as well as in T2D, and systemic inflammation is involved in development of insulin resistance in T2D. Endothelial dysfunction due to prolonged hyperglycemia is commonly seen in diabetic patients and results in micro- as well as macro-vascular and neurologic complications [1], [2], [3]. The severity of complications correlates with regulation of plasma glucose levels, i.e. to what extent the deranged metabolism is normalized [1].

Type 2 diabetes and obesity are associated with low-grade inflammation, which is particularly pronounced in visceral adipose tissue. This inflammation is believed to contribute to insulin resistance not only in the adipose tissue [4] but also in liver and skeletal muscle [5], and thereby aggravates the diabetic state. Despite the activated immune system, difficulties in clearing bacterial infections are commonly seen in patients suffering from diabetes [6], [7]. Impaired bacterial clearance is prominent during later stages of the disease and is most likely influenced by impaired peripheral blood circulation, which attenuates recruitment of leukocytes from the circulation to the infection site [8], [9]. To what extent hyperglycemia *per se* has direct effects on leukocyte recruitment and function is not fully established.

To initiate an inflammatory response to a bacterial infection, leukocytes have to migrate from the blood into the affected tissue. This process occurs in consecutive steps depicted in the leukocyte recruitment cascade. Bacterial components and chemokines released from activated macrophages (e.g. Macrophage Inflammatory Protein 2, MIP-2) are sequestered apically on nearby venular endothelial cells, and activate rolling leukocytes to upregulate integrins. This results in firm adhesion of leukocytes to the endothelium, followed by crawling to optimal sites for emigration and ultimately transmigration through the vessel wall [10], [11], [12], [13]. Extravasated leukocytes then chemotax towards the site of infection, where bacteria are killed by phagocytosis and reactive oxygen species generation [14]. Endothelial dysfunction caused by hyperglycemia is believed to contribute to development of secondary complications to diabetes, which might involve increased leukocyte-endothelial interactions and leukocyte recruitment. However, contradictory results of leukocyte-endothelial cell interactions during inflammation in diabetes models have been revealed, as either decreased [15], [16], [17] or increased [18] events are reported, as well as elevated levels of adhesion molecules [19].

By using two different mouse models of diabetes resulting in moderate and severe increased plasma glucose levels respectively, the present study investigates the role of hyperglycemia on leukocyte recruitment and function in an acute model of inflammation, in addition to a clinically relevant model of bacterial infection.

## Results

### Alloxan pre-treatment and high fat diet caused severely and moderately increased plasma glucose concentrations, respectively

**Figure 1:**
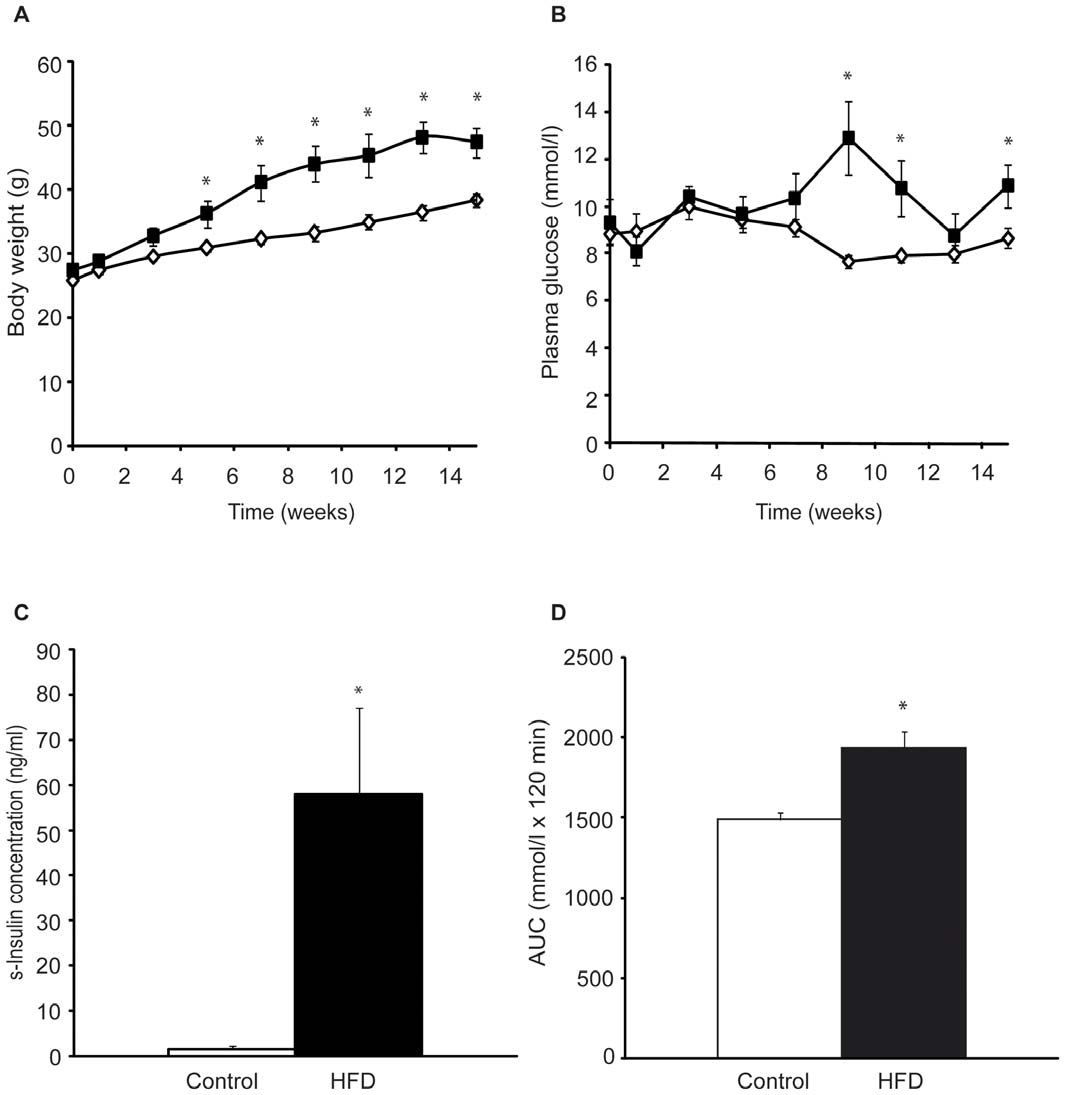
Increased weight, blood glucose and insulin, and impaired glucose tolerance in high fat diet (HFD) mice. **A**, Weight gain in control mice (white, n = 7) and mice fed a HFD (black, n = 5) and **B**, plasma glucose concentrations in mice on control (white) or high fat diet (black) over time. **C**, Serum insulin concentrations in mice after 15 weeks of control or high fat diet. **D**, Glucose tolerance test in control or high fat fed mice presented as area under curve (AUC) of plasma glucose levels×time, 15 weeks after diet start. *p<0.05. Data are means ± SEM.

Two different models of diabetes were used: one with similarities to T1D (alloxan-treatment) in which mice were severely hyperglycemic (Table 1) and insulin deficient, whereas T2D was induced by HFD which resulted in moderate hyperglycemia (Table 1) with increased serum insulin concentrations. All mice gained weight with time, but mice given a HFD had significantly higher body weights after 5 weeks on diet and onwards (Figure 1A). HFD also increased plasma glucose concentrations, resulting in significantly increased levels from 9 weeks and onwards compared to mice on control diet (Figure 1B). While serum insulin levels were undetectable in alloxan-treated mice (data not shown), HFD mice had greatly increased serum insulin levels when measured after 15 weeks of HFD (58±19 ng/ml) compared to controls (1.5±0.8 ng/ml, figure 1C). Glucose tolerance was impaired in the moderately hyperglycemic HFD mice after 15 weeks of HFD (Figure 1D).

**Table 1:** Plasma glucose levels, leukocyte rolling flux and rolling velocity in control and diabetic mice.

Plasma glucose levels (mmol/l)			
	Control	Alloxan	High fat diet
	8.5±0.5	>27.8	11.4±1.4

Rolling Flux (cells/min)			
Time (min)	Control	Alloxan	High fat diet
0	35.3±4.4	52.8±6.2	49.3±6.4
30	23.9±2.9	30.8±2.7	29.5±3.0
60	18.2±1.7	23.0±3.3	23.1±4.6
90	13.9±2.3	17.8±3.1	22.2±6.0

Rolling Velocity (µm/s)			
Time (min)	Control	Alloxan	High fat diet
0	36.9±8.9	30.6±3.6	25.5±6.5
30	37.9±8.4	37.2±4.4	32.4±9.4
60	35.7±6.6	36.5±5.8	29.7±8.5
90	33.9±6.5	31.7±3.3	30.4±8.7

Plasma glucose levels at the time of experiment, number of rolling leukocytes in the cremaster muscle per minute (Rolling flux), and the rolling velocity of the first 10 rolling leukocytes at basal level (time 0) and after 30, 60 and 90 min of MIP-2 superfusion in control (n = 6), alloxan- (n = 6) and diet-induced diabetic (n = 5) mice.

### Unstimulated leukocyte recruitment was increased in both alloxan- and diet-induced diabetes

**Figure 2:**
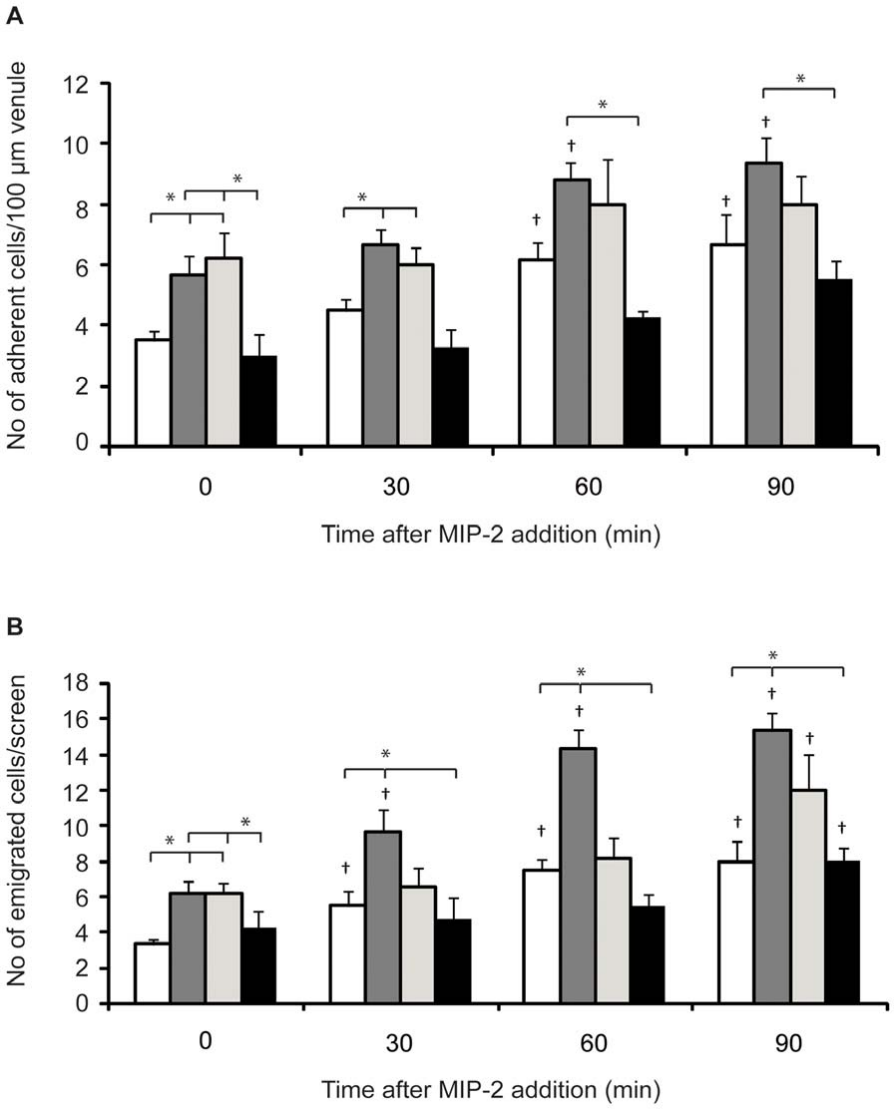
Increased leukocyte recruitment to MIP-2 in diabetic mice. The number of adherent (**A**) and emigrated cells (**B**) in the cremaster muscle of control (white, n = 6), alloxan-treated (dark grey, n = 6), high fat diet-induced diabetic (light grey, n = 5) and acute glucose-infused (black, n = 4) mice before (time 0) and after MIP-2 addition to the superfusate (0.5 nmol/L). *p<0.05 compared to other groups at the same time point. †p<0.05 compared to time 0 in the same group. Data are means ± SEM.

Leukocyte recruitment was studied using intravital microscopy of the cremaster muscle in severely hyperglycemic alloxan-treated mice (3 days after alloxan injection), mice on HFD (moderate hyperglycemia) and normoglycemic untreated mice (Table 1). At basal conditions, prior addition of the inflammatory chemokine MIP-2 to the superfusate, increased numbers of adherent and emigrated leukocytes were detected in both alloxan- and HFD-treated groups compared to control mice (Figure 2A–B). No differences in number of rolling leukocytes or their rolling velocity between these groups could be detected (Table 1).

To investigate if increased basal leukocyte recruitment in diabetic mice could be induced by acute elevation of plasma glucose concentration, a group of normoglycemic mice were pretreated with D-mannuheptulose intraperitoneally. This treatment inhibits glucose oxidation and, together with a glucose injection i.v., resulted in prolonged hyperglycemia with plasma glucose levels of 17.3±1.8 mmol/l approximately 2 h post administration. However, this acute induction of hyperglycemia did not increase the number of adherent or emigrated leukocytes compared to what was observed in untreated mice (Figure 2A–B).

These results indicate a low grade activation of the immune system and/or endothelium in mouse models of diabetes, resulting in increased number of adherent leukocytes to the postcapillary venules and ultimately increased leukocyte recruitment to tissue which is not attributable to acute increases in blood glucose levels.

### Increased leukocyte adhesion and emigration in response to MIP-2 was observed in sustained hyperglycemic mice

To investigate the role of hyperglycemia for leukocyte recruitment during inflammation, the chemokine MIP-2 was added to the superfusate of the cremaster preparation. This chemokine is known to recruit predominantly neutrophils but also some monocytes (88% versus 12% respectively) [13]. The number of rolling leukocytes decreased with time of MIP-2 superfusion to similar levels in all groups, while rolling cell velocities were not affected (Table 1), as shown previously in MIP-2 treated C57Bl/6 mice [13]. The difference in number of adherent leukocytes between normo- and hyperglycemic mice seen under basal conditions disappeared after MIP-2 addition as the number of adherent leukocytes increased to similar levels (Figure 2A). MIP-2 caused an increase of the number of emigrated cells with time in the normoglycemic mice (Figure 2B), which was also seen in both models of diabetes, but occurred at different times as the number of emigrated leukocytes in the alloxan-diabetic mice increased already after 30 min (as in normoglycemic mice), whereas the number of emigrated leukocytes in the HFD-treated mice increased only after 90 minutes of MIP-2 superfusion (Figure 2B). At the latter time point, significantly more recruited leukocytes were observed in the hyperglycemic mice compared to the normoglycemic individuals (Figure 2B), even though it did not reach significance in the diet induced model (*p* = 0.061). This indicates that leukocytes are more efficient in emigrating out of the inflamed blood vessel in the diabetic mice compared to control mice since similar levels of adherent leukocytes were observed in all groups. MIP-2 had similar effects on adherent and emigrated cells in acute hyperglycemia (D-mannuheptulose-treated) as in control mice indicating that acute hyperglycemia does not increase leukocyte recruitment during inflammation.

### The differential count is unchanged by alloxan treatment

To exclude the possibility that the observed increased leukocyte recruitment in diabetic mice was caused by change of the total number and/or proportion of immune cells in the alloxan-treated hyperglycemic model, total white blood cells and a differential count was performed. No significant differences could be detected in total white blood cells (5.4±1.9×10^9^/l and 3.1±0.5×10^9^/l), lymphocytes (84±2% and 75±5%), monocytes (9±1% and 13±3%) or granulocytes (7±1% and 12±3%) between the control (n = 4) and alloxan-treated (n = 3) mice, respectively.

### Faster initial reduction in bacterial number but impaired long-term bacterial clearance in alloxan-induced diabetic mice

**Figure 3:**
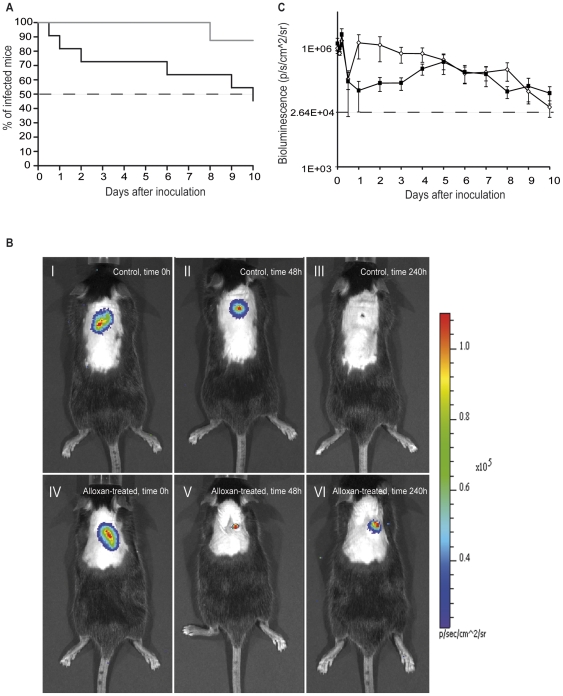
Impaired bacterial clearance in alloxan-induced diabetic mice. **A**, Clearance rate of bioluminescent *Staphylococcus aureus* (1×10^7^ CFU/ml) in control (black, n = 11) and alloxan-induced diabetic mice (grey, n = 9) until 50% of the control mice were cleared (dashed line). **B**, Representative images of bioluminescent *S. aureus* infected regions in control (upper panel) and alloxan-induced diabetic mice (lower panel) directly post inoculation (I and IV), and 2 (II and V) and 10 days (III and VI) post inoculation. **C**, Total bioluminescence in control (black, n = 11) and alloxan-treated (white, n = 9) mice over time. The dashed line represents average background bioluminescence. Data are means ± SEM.

**Figure 4:**
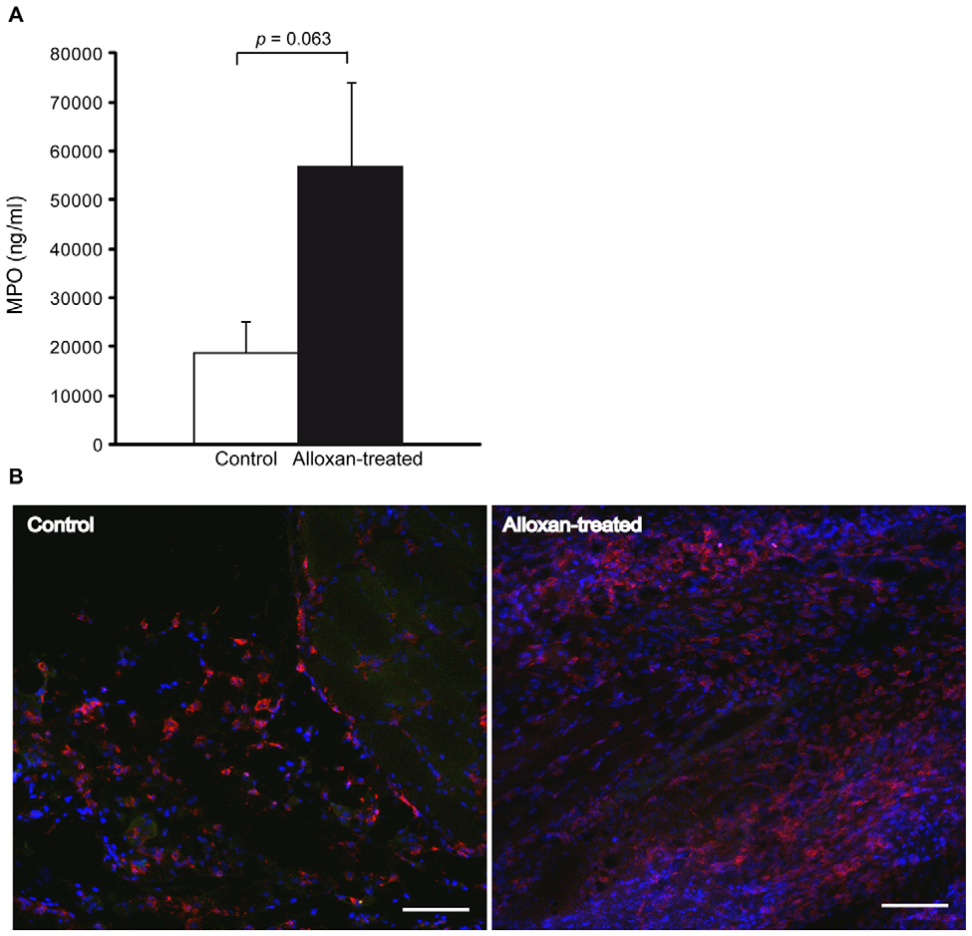
Recruited neutrophils and macrophages to the bacterial infection in control and alloxan-induced diabetic mice. Extravasated neutrophils expressed as concentration of myeloperoxidase (MPO) (**A**) in the bacteria-infected backs in control (n = 7) and alloxan-treated diabetic (n = 7) mice. Data are means ± SEM. **B**, Representative images of macrophages (Alexa555-konjugated F4/80 antibody) in bacteria-infected area in control and alloxan-treated mice. Scale bar: 100 µm.

To investigate if the observed increase in leukocyte recruitment could be transferred into a clinically relevant model of bacterial infection, normo- and hyperglycemic mice were challenged by subcutaneous *Staphylococcus aureus* (xen29, producing luciferase). Since most profound changes on leukocyte recruitment was found in the severely hyperglycemic model (alloxan-induced), this model was chosen for evaluation of bacterial clearance. The hyperglycemic mice showed decreased ability to clear the bacterial infection (Figure 3A–B) and only 12% of the alloxan-treated mice were cleared after ten days, while more than 50% of control mice were free of infections (Figure 3A). Interestingly, total bioluminescence decreased faster during the first week of infection in the alloxan-treated compared to control mice (Figure 3C) but did not disappear in any of the alloxan-treated mice during the first week (Figure 3A). The subtypes of recruited leukocytes were evaluated 2 days after bacterial infection when the inflammation was most prominent. MPO contents revealed increased neutrophil recruitment to the infected area in alloxan-treated mice, however not statistically significant (*p* = 0.063, Figure 4A), in agreement with Figure 2B. Confocal visualization of bacteria infected regions of control and alloxan-treated mice revealed presence of numerous macrophages in both groups (Figure 4B). Macrophage populations were not quantified since the exact location of the stained section in relation to the infection, which most probably reflects on number of immune cells, was difficult to appreciate.

Time with hyperglycemia might influence recruitment of leukocytes as well as their function. The type 1 diabetic mice exhibiting increased leukocyte recruitment (Figure 2) received alloxan 3 days prior experiment. The diabetic mice challenged with bacteria 3 days after alloxan treatment, were hyperglycemic for the studied period of 13 days in total (Figure 3). To exclude that the impaired bacterial clearance observed in diabetic mice was not due to decreased leukocyte recruitment at later time points, the cremaster muscle of mice hyperglycemic for 8 days was superfused with MIP-2. However, no difference could be detected in mice treated with alloxan three (n = 6) or eight (n = 7) days earlier in terms of number of recruited neutrophils after 90 min of MIP-2 superfusion: adherent cells (9.3±0.9 and 10.9±1.3), emigrated cells (15.3±1.0 and 12.9±1.4), respectively.

### Less phagocytic cells in alloxan-treated mice

**Figure 5:**
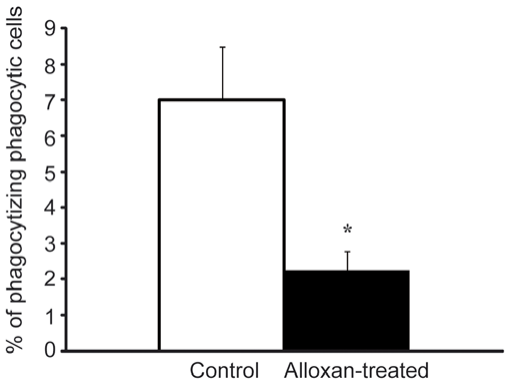
Decreased phagocytic ability of leukocytes in diabetic mice. Percentage of phagocytic leukocytes phagocytizing LPS-coated fluorescent beads in the peritoneal cavity in control (n = 5) and alloxan-treated diabetic (n = 4) mice. *p<0.05. Data are means ± SEM.

Impaired leukocyte function could explain the contradictory results of increased recruitment of leukocytes and defect bacterial clearance observed in alloxan-diabetic mice. The phagocytic ability of leukocytes was therefore examined using LPS-coated beads to mimic leukocyte activation in bacterial infection [20]. Three hours following i.p injection of LPS-coated beads, peritoneal leukocytes were isolated and analyzed for fluorescence (as a measure of phagocytosis of beads) using flow cytometry. While the populations of leukocytes with phagocytic abilities (neutrophils and macrophages) were similar (2.1±0.5 10^5^ and 2.4±0.3 10^5^ cells/ml, respectively) in the peritoneal lavages of alloxan-treated (3 days prior experiments, n = 4) and control (n = 5) mice, the number of cells that contained LPS-coated beads was decreased by 50% in the alloxan-treated mice compared to control mice (Figure 5), demonstrating impaired phagocytosis.

## Discussion

The present study investigates a common complication to diabetes, impaired bacterial clearance, using *in vivo* models of longitudinal visualization of subcutaneous infection, leukocyte recruitment in single venules and leukocyte phagocytic ability. Increased leukocyte recruitment was observed during basal conditions as well as during acute inflammation in mice with alloxan- or diet-induced diabetes, i.e. animals with severe or moderate hyperglycemia. To understand how this correlates to difficulties in clearing bacterial infections, alloxan-induced diabetic mice were challenged by *Staphylococcus aureus*. Interestingly, despite increased numbers of recruited leukocytes, diabetic mice remained infected for a longer time period compared to control mice, which correlated to a 50% decreased phagocytic ability of diabetic leukocytes.

Type 1 and type 2 diabetes are both associated with altered immune responses to bacterial infections. This is believed to be caused in part by impaired peripheral blood circulation which develops with time of fluctuating and uncontrolled plasma glucose levels [1]. The effect of hyperglycemia *per se* on leukocyte recruitment to inflamed muscle was in the present study investigated by intravital microscopy of single venules. We found that during basal conditions prior to addition of chemokines, an increased number of leukocytes were recruited into muscle in long-term but not acutely hyperglycemic mice. This might reflect that prolonged hyperglycemia induces expression of adhesion molecules on muscle venular endothelium and/or circulating leukocytes. Still more leukocytes were recruited during inflammation (MIP-2 activation) in the alloxan-induced, type 1 diabetes model, and a similar trend was seen also in the type 2 diabetes model. However, no effects were seen after an acutely induced hyperglycemia. Thus, our results show that hyperglycemic mice with time develop a more activated immune system during both non-inflammatory as well as inflammatory conditions. These effects correlate with levels of hyperglycemia, as they were more profound in the model of severe hyperglycemia even though the absence of anti-inflammatory insulin in the alloxan-treated model might contribute to this observation. Our observations are in agreement with a study of leukocyte-endothelial interactions in the mesentery during ischemia-reperfusion injury in streptozotocin-treated rats [18]. In this study, ischemia-reperfusion caused increased leukocyte adhesion and emigration to a greater extent in the diabetic rats compared to control rats, even though no differences in the number of adherent and emigrated leukocytes were seen under basal conditions prior injury between groups. However, in another study of alloxan-treated rats, a decrease in leukocyte-endothelial cell interactions in the internal spermatic fascia after inflammatory challenge was demonstrated [17]. An important discrepancy between the former study in alloxan-treated rats and the present study in mice is the time period of severe hyperglycemia, as longer hyperglycemic periods correlate with higher degree of for instance protein glycosylation that in turn might impair immune responses as well as other body functions. In our study, mice had been hyperglycemic (>27.8 mmol/l) for only 3–8 days prior the inflammatory challenge, while the rats had been confirmed hyperglycemic (>30 mmol/l) for 33 days after alloxan injection [17].

To expand our results of acute leukocyte recruitment, bacterial clearance was studied in a clinically relevant infection model in severely hyperglycemic mice, in which the leukocyte recruitment effect was most prominent. We found that the alloxan-treated mice showed decreased ability to totally clear the bacterial infection compared to control mice, despite a greater initial decrease of bacteria post-infection and increased number of recruited neutrophils to the infected area (MPO, *p* = 0.063). These results are in line with clinical findings in diabetic patients, as bacterial infections are abundantly occurring complications of the disease [6], [7], and are in accordance with a previous experimental study of *S. aureus* injected in the hindpaw of non-obese diabetic mice (NOD, an autoimmune mouse model of type 1 diabetes) [21]. However, NOD mice display numerous other immune abnormalities such as autoimmune thyroiditis, autoimmune peripheral polyneuropathy as well as systemic lupus erythematosus-like disease [22] which most probably also affect inflammatory responses needed for bacterial clearance, thus influencing the results of this study. Impaired bacterial clearance in NOD mice was attributed to decreased tissue levels of MIP-2 at the site of infection, resulting in decreased leukocyte recruitment [21]. However, whether this defect in MIP-2 secretion is due to the diabetic stage or the immune defect in NOD mice is uncertain.

The initial drop of bacterial luminescence observed in the diabetic mice could reflect increased numbers of recruited leukocytes, or an increased ability to initially kill inoculated bacteria through secretion of higher levels of toxic oxygen radicals. The latter has previously been reported in several studies. Neutrophils from diabetic cats were observed to secrete more toxic oxygen radicals when activated by phorbol myristate acetate compared to neutrophils from healthy cats [23], and intraperitoneal macrophages from alloxan-induced diabetic mice produced increased levels of reactive oxygen intermediates [24]. Further, an acute glucose challenge of healthy human subjects was shown to induce reactive oxygen species generation in neutrophils [25], and neutrophils isolated from patients with type 2 diabetes were found to produce increased amounts of reactive oxygen species compared to neutrophils from healthy controls [26]. These data cannot explain the decreased ability to clear bacterial infections shown clinically as well as in the present study. Instead, impaired bacterial clearance might be due to impaired ability to ingest, kill and remove bacteria and debris accumulated during infection. Indeed, impaired leukocyte phagocytosis has been observed in patients suffering from diabetes [27], [28]. This defect was seen already at moderately increased hyperglycemia (>11 mmol/l) indicating that it appears in patients suffering from both uncontrolled type 1 and type 2 diabetes. In the present study, we developed a phagocytic assay where leukocytes were recruited to the peritoneal cavity by injection of LPS-coated fluorescent beads, and we could detect, by using flow cytometry, a 50% decrease of leukocyte phagocytosis in alloxan-treated diabetic mice. However, these experimental results need to be confirmed in studies of leukocyte phagocytic abilities in diabetic patients suffering from recurrent bacterial infections.

In conclusion, the results presented here suggest that the reduced ability to clear bacterial infections during diabetes is not due to impaired leukocyte recruitment as prolonged hyperglycemia caused increased number of emigrated leukocytes in tissue during basal conditions as well as during acute inflammation. Despite the increased numbers of recruited leukocytes to inflammation in the diabetic mice, the bacterial infection remained for longer periods due to a defect leukocyte phagocytosis.

## Materials and Methods

### Ethical statement

All experiments were approved by the Swedish Laboratory Animal Committee in Uppsala, approval number C268/9 and C180/10.

### Animals

C57BL/6 male mice, (B&K Universal, Scanbur, Stockholm, Sweden), were housed in animal facility under standardized conditions (21–22°C, 12 h light/12 h darkness) with free access to tap water and pelleted food (Type R36, Lantmännen, Kimstad, Sweden) or high fat diet (HFD; 60% kcal from fat, D12492; Research diets, New Brunswick, NJ, USA).

### Induction of hyperglycemia

Acute hyperglycemia: Acute and prolonged hyperglycemia was induced by intraperitoneal injection of D-Mannuheptulose (a glucose oxidation inhibitor, 0.2 µg/kg body weight, Sigma-Aldrich, St. Louis, MO, USA), before intravenous glucose injection (0.6 g/kg b.w). Type 1 diabetes model: Hyperglycemia (plasma glucose levels >27.8 mmol/l) was induced by a tail vein injection of alloxan (75 mg/kg body weight, Sigma-Aldrich) [29], [30], a β-cell toxin, 3 or 8 days before intravital microscopy experiment or inoculation of bacteria. Type 2 diabetes model: Moderate hyperglycemia (plasma glucose levels ∼11 mmol/l) was induced by HFD for 15 weeks.

### Plasma glucose and serum insulin concentrations

Plasma glucose levels were monitored in blood from the tail with test reagent strips (detection range 1.1–27.8 mmol/l; Freestyle, Abbott, Stockholm, Sweden) once a week (control and HFD mice) and daily following alloxan administration. Serum insulin concentrations were measured with insulin ELISA (detection limit 1.5 µg/l, Mercodia Rat Insulin ELISA; Mercodia AB, Uppsala, Sweden) in mice after 15 weeks of HFD.

### Glucose tolerance tests

Glucose tolerance test was performed after 15 weeks of HFD and in aged-matched controls. Plasma glucose concentrations in blood from the tail were measured with test reagent strips before administering 2 g/kg body weight D-glucose (300 mg/ml; Fresenius Kabi, Uppsala, Sweden) intravenously into the tail. Plasma glucose concentrations were thereafter measured at 10, 30, 60, and 120 min post-administration.

### In vivo recruitment of leukocytes

Aged-matched mice were anaesthetized by spontaneous inhalation of ∼2.4% isoflurane (Forene®, Abbott Scandinavia AB, Stockholm, Sweden) in a mixture of air and oxygen (total oxygen 40%) through a breathing mask connected to an isoflurane pump (Univentor 400 Anesthesia Unit, AgnTho's AB, Lidingö, Sweden). Plasma glucose concentrations were measured before and after induction of anesthesia, and the levels were not affected by isoflurane anesthesia. The animals were placed on a water-heated operating table to maintain body temperature at ∼37°C. The depth of anesthesia was controlled by regularly monitoring peripheral reflexes. The cremaster muscle was prepared as previously described [31], [32]. Briefly, the muscle was dissected free and opened longitudinally with cautery. It was held flat on cover slip by attaching five sutures in the corners and was then constantly superfused (1 ml/min) with pre-warmed bicarbonate buffered saline (pH 7.4), throughout the experiment. After a 30-minute resting period, a cremasteric venule with a diameter of ∼25–35 µm was selected for blood flow recording during a 5-minute-period through an intravital microscope (Leica Microsystems DM5000B, Wetzlar, Germany) with a CCD camera (Hamamatsu Orca-R2, Hamamatsu City, Japan) connected to a computer with Volocity 5.0 Acquisition software. After the first recording period, the chemokine MIP-2 (CXCL2; R&D Systems, Abingdon, UK) was added to the superfusate throughout the remaining experiment (0.5 nmol/l). MIP-2, the murine homologue to IL-8, binds to the receptor CXCR2 and recruits predominantly neutrophils [31]. Five minute periods were recorded at 30, 60 and 90 min after MIP-2 addition. The number of rolling leukocytes was counted during each of these 5-minute-periods and average number of rolling leukocytes per min was calculated. The rolling cell velocity of the first ten rolling cells during each period was measured. Also, the number of adherent leukocytes in a 100 µm long segment of the venule, as well as the number of emigrated cells in the field of view (200 µm×300 µm, 0.06 mm^2^) was analyzed. The experiments were ended by collecting blood through heart puncture for analysis of serum insulin concentrations.

### Differential counts

Blood from alloxan-treated and control mice were drawn via heart puncture into Microtainer K2E tubes (BD Franklin Lakes, USA) for white blood cell differential count using an ABX MICROS 60 CS/CT, ABX Diagnostics (Montpellier, France).

### Bacterial clearance imaging

The mice had their fur on their backs removed using shaver and depilation cream (Veet, Reckitt Benckiser, Valora Trade, Stockholm, Sweden) 24 h prior to bacterial inoculation. Bioluminescent *Staphylococcus aureus* (Xen29, Caliper life sciences; Alameda, USA) cultured overnight in liquid medium (10 ml LB medium with 200 µg/ml kanamycin) was subcultured (1∶10) until exponential growth rate (approximately 1 h). The amount of bacteria was determined using spectrophotometry (OD_600_ = 0.5∼1.4×10^8^ CFU/ml). ∼1×10^7^ CFU were suspended in PBS solution containing 10 mg/ml of inert Cytodex beads (Sigma Aldrich). Approximately ∼1×10^6^ CFU bacteria in 100 µl PBS solution were inoculated subcutaneously in anaesthetized alloxan-treated (plasma glucose >27.8 mmol/l) or normoglycemic mice. Bacterial infection was monitored using a bioimaging device (IVIS Spectrum, Caliper Life Sciences, Hopkinton, USA) in isoflurane anaesthetized mice at time points 0, 2, 5, 12, and 24 h, and then every 24 h post inoculation for 10 days. Images were analyzed and bio-luminescence quantification was done using Living Image 3.1 software (Caliper).

### In vitro evaluation of inflammation

Some infected mice were sacrificed two days after subcutaneous bacteria inoculation to evaluate type and amount of recruited leukocytes. The center of infected backs was collected and tissue samples (∼20 mg) were homogenized in lysis buffer and analyzed for Myeloperoxidase (MPO) content using mouse MPO ELISA (Hycult biotech, Uden, Netherlands). The remaining tissue samples were mounted in tissue tek (Cellab, Sollentuna, Sweden), frozen in −70°C prior cryosectioning and staining of macrophage content using anti-F4/80 antibody (conjugated with Alexa Fluor 555) and nuclei using Hoechst 33342 (Invitrogen, Stockholm, Sweden) before confocal microscopy (Nikon C-1 with Plan Fluor ELWD 20×/0.45 objective) analysis.

### Phagocytosis assay

Fluorescent beads (2 µm, Polyscience, Inc., Eppelheim, Germany) coated with LPS (lipopolysaccharide, 100 µg/ml, Sigma-Aldrich) at 4°C over night were injected intraperitoneally in mice treated with alloxan 3 days earlier or control mice. Three hours later, recruited phagocytic cells were isolated via peritoneal lavage in isoflurane-anesthetized mice. Four ml of HBSS (Sigma-Aldrich) were injected into the peritoneal cavity followed by a 5-minute massage of the abdomen before the fluid was collected. Leukocytes were isolated via Percoll gradient isolation consisting of Percoll stock solution (9 ml of Percoll and 1 ml of 10× PBS) diluted into 72%, 64%, and 52% in PBS [33]. The total populations of phagocytic leukocytes as well as percentage of the population that contained fluorescent beads were determined using flow cytometry (FACScalibur, BD Biosciences). Data are presented as percentage of phagocytic cells that contained beads.

### Statistical analysis

All values are expressed as mean ± SEM. One way repeated measurements of ANOVA was used when comparing the same animal at different time points with Dunnett's post hoc test. To compare groups at the same time point, one way ANOVA with Fischer's LSD post hoc test was used. Student's t-test was used when two groups were compared. Statistical significance was set to P<0.05. All statistical analyses were carried out using SigmaStat 3.5 (Systat Software, Richmond, VA, USA) except the Kaplan-Meier test performed in GraphPad Prism 5 (GraphPad Software Inc, La Jolla, CA, USA).
